# Supplementary material for: Freshwater monitoring by nanopore sequencing
Source: eLife. 2021 Jan 19;10:e61504. doi: 10.7554/eLife.61504 (PMC7815314; doi:10.7554/eLife.61504)
Supplement: Transparent reporting form [file elife-61504-transrepform.pdf]

eLIFE
1st Floor
24 Hills Road
Cambridge CB2 1JP, UK
P 01223 855340
W elifesciences.org
T @elife

eLife's transparent reporting form

We encourage authors to provide detailed information *within their submission* to facilitate the interpretation and replication of experiments. Authors can upload supporting documentation to indicate the use of appropriate reporting guidelines for health-related research (see EQUATOR Network), life science research (see the BioSharing Information Resource), or the ARRIVE guidelines for reporting work involving animal research. Where applicable, authors should refer to any relevant reporting standards documents in this form.

If you have any questions, please consult our Journal Policies and/or contact us: editorial@elifesciences.org.

Sample-size estimation

- You should state whether an appropriate sample size was computed when the study was being designed
- You should state the statistical method of sample size computation and any required assumptions
- If no explicit power analysis was used, you should describe how you decided what sample (replicate) size (number) to use

Please outline where this information can be found within the submission (e.g., sections or figure legends), or explain why this information doesn't apply to your submission:

No explicit power analysis was used in our proof-of-principle study.

Barcoded MinION sequencing was done on three separate flowcells, each with approximately equimolar inputs of:
- DNA extracts from 9 river locations (+ 1 biological replicate of the same location)
- 1 negative control (DNA from deionised water)
- 1 mock community (DNA from Zymo D6305)
= 3 x 12 = **36 samples** of full-length 16S nanopore sequencing

All but the mock community isolates were also submitted for ICP-OES and chromatography trace ion measurements, as well as Gold Standard Leptospira qPCR at Public Health England.

Detailed summary illustrations and descriptions of our samples can be found in the manuscript's **Fig. 1, Supplementary Files 1 and 2, Material and Methods sections 1.1-1.4**.

Replicates

- You should report how often each experiment was performed
- You should include a definition of biological versus technical replication
- The data obtained should be provided and sufficient information should be provided to indicate the number of independent biological and/or technical replicates
- If you encountered any outliers, you should describe how these were handled
- Criteria for exclusion/inclusion of data should be clearly stated
- High-throughput sequence data should be uploaded before submission, with a private link for reviewers provided (these are available from both GEO and ArrayExpress)

eLife Sciences Publications, Ltd is a limited liability non-profit non-stock corporation incorporated in the State of Delaware, USA, with company number 5030732, and is registered in the UK with company number FC030576 and branch number BR015634 at the address 1st Floor, 24 Hills Road, Cambridge CB2 1JP | August 2014

Please outline where this information can be found within the submission (e.g., sections or figure legends), or explain why this information doesn't apply to your submission:

Major sections of our manuscript are devoted to the determination of the statistical robustness of the workflow in context of spatiotemporal river monitoring. We have consequently included several control samples (see above), software assessments across the same read data (Figure 2, Figure 2–figure supplement 1), downsampling tests and stringent sample exclusion (Figure 3, Figure 3–figure supplement 1, Figure 5–figure supplement 1a-b).

Most details on replicate usage, sequencing data inclusion, downsampling and deposition are provided in **Figure 2, Figure 2–figure supplement 1, Figure 3**, within their corresponding **figure legends**, as well as **Material and Methods sections 2.4, 2.6.**, and **Supplementary File 1a** (for sample-wise ENA identifiers).

Statistical reporting

- Statistical analysis methods should be described and justified
- Raw data should be presented in figures whenever informative to do so (typically when N per group is less than 10)
- For each experiment, you should identify the statistical tests used, exact values of N, definitions of center, methods of multiple test correction, and dispersion and precision measures (e.g., mean, median, SD, SEM, confidence intervals; and, for the major substantive results, a measure of effect size (e.g., Pearson's r, Cohen's d)
- Report exact p-values wherever possible alongside the summary statistics and 95% confidence intervals. These should be reported for all key questions and not only when the p-value is less than 0.05.

Please outline where this information can be found within the submission (e.g., sections or figure legends), or explain why this information doesn't apply to your submission:

Details on statistical treatments, sample groups and significances form part of the **legends of results Figures 2, Figure 2–figure supplement 1, Figure 3, Figure 3–figure supplement 1, Figure 4, Figure 4–figure supplement 1, Figure 5, Figure 5–figure supplement 1, Figure 6, Figure 7, Figure 8**. Additionally, clarifications on e.g. RMSE (Methods section 2.2.2) or Mantel test (Methods section 2.4.1) usage have been provided.

(For large datasets, or papers with a very large number of statistical tests, you may upload a single table file with tests, Ns, etc., with reference to sections in the manuscript.)

Group allocation

- Indicate how samples were allocated into experimental groups (in the case of clinical studies, please specify allocation to treatment method); if randomization was used, please also state if restricted randomization was applied
- Indicate if masking was used during group allocation, data collection and/or data analysis

Please outline where this information can be found within the submission (e.g., sections or figure legends), or explain why this information doesn't apply to your submission:

Following our sequencing approach at three separate time points/batches, our figures and tables reflect these three data groups in terms of their colouring and structuring:
- Figure 4a, Figure 4–figure supplement 4a, Figure 5a, Figure 5–figure supplement 1a-b Figure 6b (April = green, June = orange, August = blue)
- Figure 2–figure supplement 2, Figure 7, Figure 8 (grey scaling between April, June, August)
- Supplementary File 1b-c, Supplementary File 2, Supplementary File 5 (rows ordered by April, June, August)
- Supplementary File 8 (separate tabs for April, June, August)

On a lower hierarchy level, we also maintain a consistent colouring for each sampling location in Figure 1, Figure 1–figure supplement 1, Figure 3a, Figure 3–figure supplement 1, Figure 4a, Figure 4–figure supplement 1a, Figure 6c, Figure 7, Figure 8 (from 1 = dark blue, to 9 = dark red)

Asides from these display arrangements and read pooling in Figure 4b and Figure 4–figure supplement 1b (described in the corresponding legends), the **sample data was treated fully independently** across all analyses.

Additional data files ("source data")

- We encourage you to upload relevant additional data files, such as numerical data that are represented as a graph in a figure, or as a summary table
- Where provided, these should be in the most useful format, and they can be uploaded as "Source data" files linked to a main figure or table
- Include model definition files including the full list of parameters used
- Include code used for data analysis (e.g., R, MatLab)
- Avoid stating that data files are "available upon request"

Please indicate the figures or tables for which source data files have been provided:

Sequencing datasets generated and analysed during this study are available from the European Nucleotide Archive (https://www.ebi.ac.uk/ena/data/view/PRJEB34900). The following figures of this manuscript are based on this data: Figures 2, 3, 4, 5, 7, 8, Figure 2–figure supplement 1, Figure 2–figure supplement 2, Figure 3–figure supplement 1, Figure 4–figure supplement 1, Figure 5–figure supplement 1.

Environmental measurements are available from public repositories, https://www.cl.cam.ac.uk/research/dtg/weather/ and https://nrfa.ceh.ac.uk/. The following figures of this manuscript are based on this data: Figure 6 and Figure 6–figure supplement 1.

Our Github repository (https://github.com/d-j-k/puntseq/) features a Snakemake framework that integrates all data pre-processing steps, and a Singularity that contains all necessary software (https://github.com/d-jk/puntseq/tree/master/analysis/). We further provide complete and rarefied SILVA 132 classifications from runs of Minimap2 (https://github.com/d-j-k/puntseq/tree/master/minimap2_classifications), which can be directly used as an input for reproducible downstream analyses.

There are **no restrictions on data or code availability**.
